# LAMP: disease classification derived from layered assessment on modules and pathways in the human gene network

**DOI:** 10.1186/s12859-020-03800-2

**Published:** 2020-10-30

**Authors:** Zhilong Mi, Binghui Guo, Xiaobo Yang, Ziqiao Yin, Zhiming Zheng

**Affiliations:** 1 Beijing Advanced Innovation Center for Big Data and Brain Computing and LMIB, Beihang University, Beijing, China; 2 Peng Cheng Laboratory, Shenzhen, Guangdong Province China; 3 School of Mathematical Sciences and Shenyuan Honors College, Beihang University, Beijing, China

**Keywords:** LAMP, Disease classification, Modules, Pathways, Human gene network, Entropy

## Abstract

**Background:**

Classification of diseases based on genetic information is of great significance as the basis for precision medicine, increasing the understanding of disease etiology and revolutionizing personalized medicine. Much effort has been directed at understanding disease associations by constructing disease networks, and classifying patient samples according to gene expression data. Integrating human gene networks overcomes limited coverage of genes. Incorporating pathway information into disease classification procedure addresses the challenge of cellular heterogeneity across patients.

**Results:**

In this work, we propose a disease classification model LAMP, which concentrates on the layered assessment on modules and pathways. Directed human gene interactions are the foundation of constructing the human gene network, where the significant roles of disease and pathway genes are recognized. The fast unfolding algorithm identifies 11 modules in the largest connected component. Then layered networks are introduced to distinguish positions of genes in propagating information from sources to targets. After gene screening, hierarchical clustering and refined process, 1726 diseases from KEGG are classified into 18 categories. Also, it is expounded that diseases with overlapping genes may not belong to the same category in LAMP. Within each category, entropy is applied to measure the compositional complexity, and to evaluate the prospects for combination diagnosis and gene-targeted therapy for diseases.

**Conclusion:**

In this work, by collecting data from BioGRID and KEGG, we develop a disease classification model LAMP, to support people to view diseases from the perspective of commonalities in etiology and pathology. Comprehensive research on existing diseases can help meet the challenges of unknown diseases. The results provide suggestions for combination diagnosis and gene-targeted therapy, which motivates clinicians and researchers to reposition the understanding of diseases and explore diagnosis and therapy strategies.

## Background

Traditionally, the classification of diseases stems from the correlation between clinical syndromes and pathological analysis. Although it has served clinicians well, it is intrinsically limited in the 21st century biological big data era [1]. Genes play a crucial role in cellular process regulation and disease development. Understanding the relationships between diseases on account of underlying biology can provide new insights into disease classification [2, 3]. The significance of redefining human disease in the era of precision medicine cannot be overemphasized [1].

The microarray technology enables people to classify diseases based on gene expression profiles. However, cellular heterogeneity within tissues and genetic heterogeneity between samples challenge the expression-based classification in complex diseases [4]. Integrating pathway information addresses these challenges [4]. Based on the associations with biological pathways, Li et al. [2] present a way of discovering relationships between human diseases, which offers novel therapeutic opportunities for medicines. By inferring pathway activities, Su et al. propose a classification method and achieve more reproducible pathway markers of breast cancer metastasis [5]. However, the pathway-based classifiers are limited to the coverage of genes by known biological pathways. To overcome this problem, one possible approach is to integrate human gene networks to overlay more genes.

The availability of gene relationships promotes the development of network medicine [6], which has the potential to indicate the complexity of diseases at the molecular level and offer computational methods for therapy strategies [7]. Pathway and network based approaches enable us to systematically explore the relationships between biomarkers and interacting molecules [8]. Combining biomedical data with networks helps to evaluate disease etiologies and identify treatment markers [9]. Disease classification is indispensable for achieving precision medicine, and associated biological pathways should be properly reflected in disease description [10]. Further, disease classification methods are expected to increase knowledge of disease etiology and revolutionize personalized medicine [3, 6].

In this work, we propose a disease classification approach focusing on the layered assessment on modules and pathways (LAMP). Directed human gene interactions are the foundation of constructing a directed human gene network (HGN). The largest connected component (LCC) contains most disease and pathway genes, of which the significant roles are recognized. The fast unfolding algorithm identifies 11 modules in LCC. Then layered networks are introduced to distinguish positions of genes in propagating information between diseases, modules and pathways. For 1726 diseases from KEGG, gene screening, hierarchical clustering and refined process result in 18 categories. After that, it is expounded that diseases with overlapping genes may not belong to the same category in LAMP. Both KEGG and LAMP classification allow us to view diseases from the perspective of commonalities in etiology and pathology. Further, entropy of KEGG and LAMP categories evaluates the prospects for combination diagnosis and gene-targeted therapy.

## Results

### Recognizing the significant roles of disease and pathway genes in the human gene network

Traditionally, the classification of diseases stems from the correlation between clinical syndromes and pathological analysis. For example, disease classification in KEGG is based on diseased tissues and organs as well as congenital factors [11, 12]. 1726 diseases are considered in this work because their genes are in the LCC. Detailed diseases information can be found in Additional file 1. The human gene network provides a perspective for studying the association between disease genes and pathway genes, enabling us to systematically assess the etiology of diseases.

**Fig. 1:**
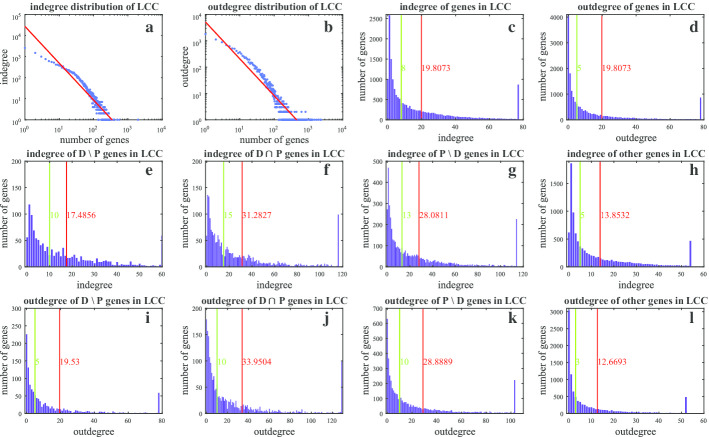
Degree distribution. **a**, **b** Dual logarithmic coordinate diagrams of indegrees and outdegrees of LCC genes. **c**, **d** Histograms of indegrees and outdegrees of LCC genes. In each panel, the red and green line represent the average and median degree, respectively. In addition, the top 5% degrees are combined into the last column. **e**–**l** Histograms of indegrees and outdegrees of D${\setminus }$P, D$\cap$P, P${\setminus }$D and other genes, respectively. ${\setminus }$ and $\cap$ are set operators. The dyadic operation between two sets A and B, say, resulting in the set A${\setminus }$B consisting of those elements that are in A but not in B. The dyadic operation between two sets A and B, say, resulting in the set A$\cap$B consisting of those elements that are in both A and B

Degree distribution reveals significant regulatory role of disease and pathway genes. The LCC indegree distribution obeys a power law distribution with $\gamma =1.7502$, and LCC outdegree distribution obeys a power law distribution with $\gamma =1.4001$, (see in Fig. 1a, b). The average indegree and outdegree of genes in LCC is 19.8073 (marked with red lines in Fig. 1c, d). The indegree is larger than the outdegree for most genes. Consistent with the above conclusions is the following fact that the median indegree is 8, which is larger than the median outdegree 5. The disease genes and pathway genes are a high-priority part of the entire network for the following facts (see in Fig. 1e–l). First, the average degrees (indegree and outdegree) and median degrees (indegree and outdegree) of disease and pathway genes are much larger than those of other genes in LCC, which emphasizes the significance of disease and pathway genes. Second, for disease and pathway genes, the median indegree is larger than the median outdegree, however, the average indegree is a little less than the average outdegree. The explication is that for disease and pathway genes, the maximum outdegree is larger than the maximum indegree, and the proportion of genes with high outdegree is larger than the proportion of genes with high indegree. In fact, for D${\setminus }$P, D$\cap$P, P${\setminus }$D genes, the maximum indegrees are 261, 489 and 1974, respectively, while the maximum outdegree are 1339, 2328 and 1938, respectively. Note that the second maximum indegree of D${\setminus }$P genes is 564. This points out that disease and pathway genes play a more extensive role compared with other genes, particularly, minorities are of significant function in gene interactions.

**Fig. 2:**
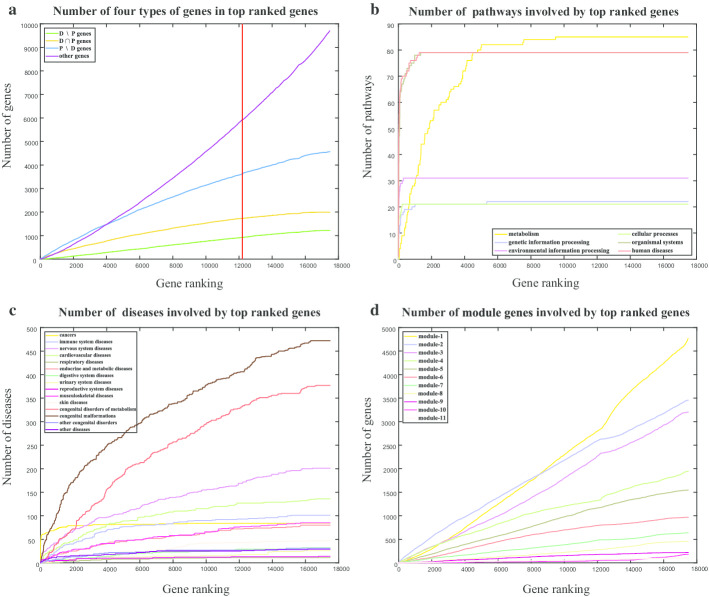
Betweenness centrality. **a** Number of four types of genes in top ranked genes. The red line represents the mark 12,198. **b** Number of pathways involved by top ranked genes. **c** Number of diseases involved by top ranked genes. **d** Number of module genes involved by top ranked genes

In addition, betweenness centrality indicates the difference between gene sets. Genes of LCC are sorted by betweenness centrality from high to low to find the top ranked genes such as APP, TP53, EGFR are very well studied disease genes [13]. 12,198 genes, almost all of which are LSCC (largest strongly connected component) genes, have a betweenness centrality larger than 0, implying that LSCC genes play a key role as a bridge in gene network regulations. Besides, disease genes and pathway genes account for the majority in the 12,198 genes (Fig. 2a). Also, all four types (D${\setminus }$P, D$\cap$P, P${\setminus }$D, and other genes) are close to evenly distributed in the list of ranked genes. When pathway genes make up the pathway as a whole, the differences come out. Metabolism pathway genes are distributed among the top 10,000 ranked genes, nevertheless, most of other 5 types pathways are involved by at least one pathway gene in top 200 (Fig. 2b). The growth trends of pathways of organismal systems and human diseases are almost the same, and the growth trends of pathways of genetic information processing and cellular processes are almost the same (Fig. 2b).

When it comes to diseases, cancers are always the first primary concern. TP53 ranked fourth in the betweenness centrality, and it is a disease gene of 42 cancers, so the number of cancers involved by top ranked genes is growing rapidly at the beginning, and is the fastest to reach the maximum (Fig. 2c). Congenital malformations diseases and congenital disorders of metabolism diseases are two major diseases due to the large numbers, but the disease genes of half these diseases ranked after 3000. The same is true for other types of diseases except cancers, which makes cancers different. Under the existing classification of diseases, even for the same group of diseases, the status of the corresponding disease genes in information transmission is very different. Developing a classification in view of the effects of disease genes on pathway functions may work well.

### Preliminary results of LAMP classification: reflecting the associations of pathways and diseases

In this work, importance of the integration of knowledge on the etiology is attached. Three modularity concepts have been reviewed including topological modules, functional modules and disease module, to help recognize the network-based position of disease genes [14]. As a result of executing the fast unfolding algorithm [15], 11 modules are identified in LCC. Figure 2d points out that all the 11 modules of genes are close to evenly distributed in the list of ranked genes, especially for those genes with positive betweenness centrality. This implies that all the modules are composed of genes of different levels, in the context of betweenness centrality.

**Fig. 3:**
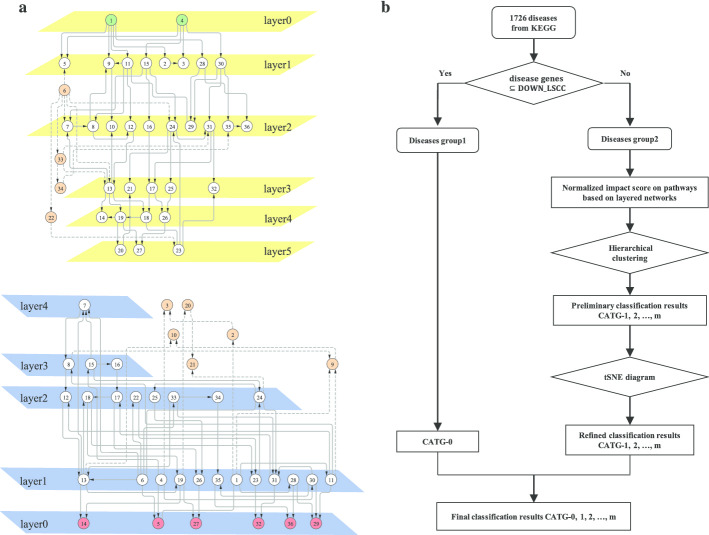
Layered networks and LAMP classification approach flow. **a** Illustration of layered networks of the original network with 36 nodes and 62 edges. The forward layered network is on the top with green nodes as the source nodes. The reverse layered network is on the bottom with red nodes as the target nodes. Orange nodes and dashed edges do not belong to these layered networks. **b** Flow diagram of the LAMP classification approach. 1726 diseases are divided into 2 group in gene screening process corresponding to whether the disease genes are all screened in DOWN_LSCC. Answer yes to get diseases group1, which are considered to belong to the same category, denoted by CATG-0. Answer no to get diseases group2. Preliminary results are obtained after hierarchical clustering. Refined results are obtained from the tSNE diagram, which together with CATG-0 constituted the final results

**Fig. 4:**
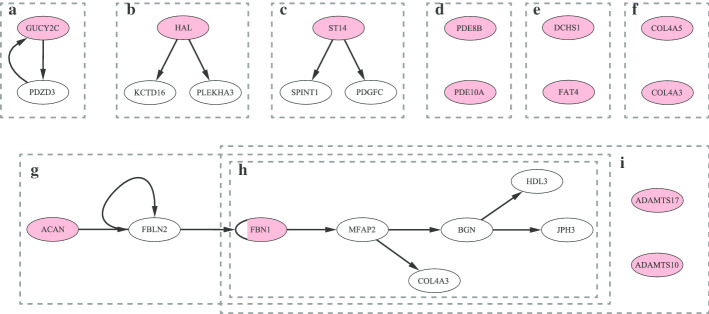
Illustration of disease genes (pink) and downstream genes (white) of the following CATG-0 diseases. **a** Irritable bowel syndrome. **b** Histidinemia. **c** Ichthyosis with hypotrichosis. **d** Autosomal dominant striatal degeneration. **e** Van Maldergem syndrome. **f** Alport syndrome. **g** Familial osteochondritis dissecans, Spondyloepiphyseal dysplasia (Kimberley type). **h** Stiff skin syndrome, Marfan syndrome, MASS phenotype. **i** Weill-Marchesani syndrome. Note that diseases in **g**, **h**, **i** share a part of disease genes and downstream genes. The different color of gene FBN1 means that it is not a disease gene in **g** but a disease gene in **h** and **i**

Then the disease classification approach is performed focusing on the layered assessment on modules and pathways. An example network and specific layered networks are illustrated in Fig. 3a, and a summary of the LAMP classification is shown in Fig. 3b (more details are available in Methods). We represent UP_LSCC the set of genes in LCC ${\setminus }$ LSCC that can access LSCC genes. Thus, there is a path from genes in UP_LSCC to genes in LSCC but no path from genes in LSCC to genes in UP_LSCC. We represent DOWN_LSCC the set of genes in LCC ${\setminus }$ LSCC that can be accessed by LSCC genes. Thus, there is a path from genes in LSCC to genes in DOWN_LSCC but no path from genes in DOWN_LSCC to genes in LSCC. The size of modules in each part of LCC can be found in Additional file 2. Implementing the maximum matching algorithm [16, 17] finds out that the 5136 of 17,486 LCC nodes are driver nodes to ensure structural controllability [17] of the linear control system, not to mention the nonlinear system. Almost $95\%$ of UP_LSCC genes are driver nodes (see in Additional file 2), which makes sense because very few genes point to them. The perturbations of UP_LSCC genes not only have wide influence but also lack internal adjustments to correct. However, the perturbation of genes in DOWN_LSCC only have an impact on DOWN_LSCC genes. Totally 440 disease genes are screened in DOWN_LSCC, which means that perturbation of the state of these genes does not affect the vast majority of genes through interactions. Further, 151 diseases caused only by the above 440 disease genes. Denoted by CATG-0 (also see in Additional file 3), we group these 151 diseases together for the reason that the targeted drug therapy may be safe with very limited side effects in the context of gene interactions. Figure 4 illustrates the disease genes (pink) and their downstream genes (white) for 12 diseases. The other 139 diseases are single-gene diseases, and there are no more downstream nodes.

**Fig. 5:**
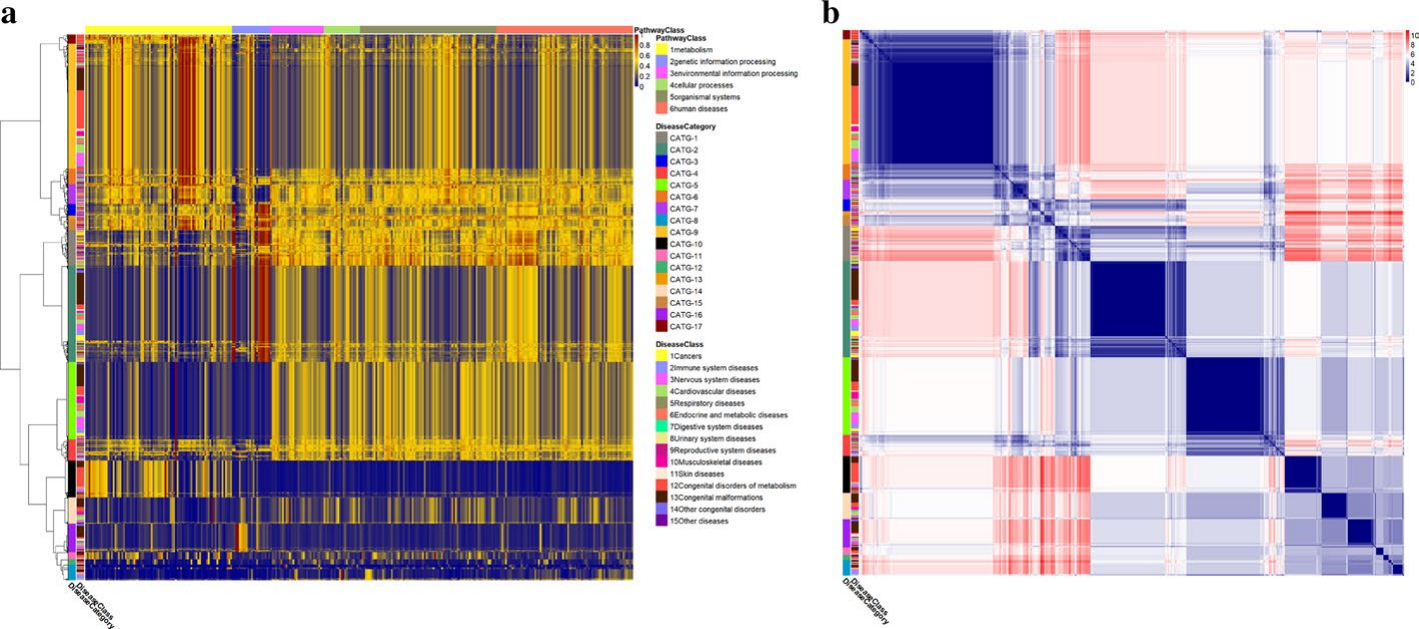
Preliminary classification results. **a** Hierarchical clustering of 1575 diseases yields 17 categories (CATG-1, 2, ..., 17). For the 1575 diseases, the normalized impact score vector $NIS(D_l)$ corresponding to pathways are calculated, and the euclidean distance is used to measure the distance between two diseases. **b** The distance matrix of 1575 diseases, which are arranged in the order in **a**

In the following, we focus on the output influence on modules and pathways from disease genes of rest 1575 diseases. The normalized impact score vectors $NIS(D_l)$ corresponding to pathways are calculated. Hierarchical clustering of 1575 diseases yields 17 categories (see Fig. 5a and Additional file 4). The distance matrix of 1575 diseases is illustrated in Fig. 5b in the clustering order in the dendrogram. Each of 17 categories contains several types of diseases in KEGG, suggesting similarities impacts on pathways. Focusing on cancers, the results show that CATG-1 and CATG-2 both possess 32 cancers. Specifically, cancers in CATG-1 are mainly composed of cancers of the digestive system and cancers of the breast and female genital organs, however, cancers in CATG-2 are mainly composed of cancers of soft tissues and bone and cancers of haematopoietic and lymphoid tissues. Some myoma diseases such as subependymal giant cell astrocytoma, lymphangioleiomyomatosis, renal angiomyolipoma, uterine leiomyoma, are also grouped in CATG-1, although the locations of the lesions are different. Cancers may occur in the same tissues, organs, and systems, but their effects on pathways also exist differences, same for the other diseases. Figure 5a illustrates that CATG-1 and CATG-2 diseases mainly affect pathways of genetic information processing, however, CATG-1 diseases also have apparent impacts on pathways of environmental information processing, cellular processes, organismal systems and human diseases.

Pathways of metabolism are less affected by diseases, except for endocrine and metabolic diseases and congenital disorders of metabolism. In detail, most endocrine and metabolic diseases are grouped in CATG-9, which mainly affect biosynthesis pathways of steroid, mannose type O-glycan, etc. Most congenital disorders of metabolism are grouped in CATG-10, which mainly affect metabolism pathways of arachidonic acid, linoleic acid, nicotinate, nicotinamide, and retinol, as well as degradation pathways of glycosaminoglycan and other glycans. A small part of congenital disorders of metabolism are grouped in CATG-17, which affect metabolism pathways relatively evenly.

Note that CATG-4 and CATG-5 diseases mostly affect D-Arginine and D-ornithine metabolism pathway, the only gene in which is DAO (D-amino acid oxidase). Alzheimer’s disease (AD) is a typical example in CATG-5. More and more researches are conducted to explore the relationship between AD and D-amino acid level alterations. Fisher et al. show that the degenerative process in the brain can be reflected by the higher concentrations of D- and L-amino acids in AD ventricular cerebrospinal fluid [18]. Lin et al. indicate that age-related cognitive declines while the peripheral DAO levels increase [19]. Autism and schizophrenia are typical examples in CATG-4. Chung et al. indicate significant associations between SNPs of the DAO gene and boys with autism spectrum disorders [20]. Chumakov et al. reveal the association of both DAO and G72 with schizophrenia [21]. In recent years, trials of sodium benzoate, a D-amino acid oxidase inhibitor, have been contacted for the treatment of mild AD [22], autism [23], schizophrenia [24], resulting in symptomatology improvement of patients. D-amino acids have important functions in the nervous system and DAO is associated with microbial induction of intestinal [25]. Recent work has suggested that brain function can be affected by microbiota in healthy and diseased individuals [26]. Based on the associations between nervous system, D-amino acids and microbiota, we agree that novel methods for treating neurological diseases could be suggested by studying microbiota-gut-brain axis mechanisms [26].

In addition, CATG-16 diseases mostly affect spliceosome pathway, followed by ribosome pathway, mRNA surveillance pathway, ribosome biogenesis pathway. In this case, genetic information processing should be severely affected. As expected, most diseases in CATG-16 are congenital malformations, congenital disorders of metabolism or other congenital disorders. Prevention and treatment of congenital disorders are of equal importance.

### Refined results of LAMP classification: revealing the multiple attributes of diseases

**Fig. 6:**
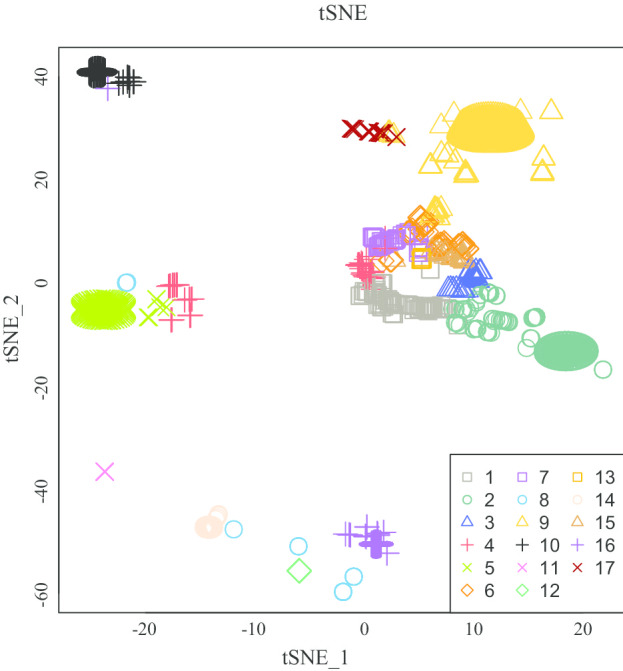
Visualization in the tSNE diagram. Visualization of the normalized impact score of 1575 diseases in a two-dimensional tSNE diagram

A two-dimensional tSNE diagram [27] is drawn to visualize the results of 17 categories, verify and refine the disease classification (see in Fig. 6). The tSNE function is a nonlinear embedding technique that is commonly used for finding a faithful representation of high-dimensional data in a lower-dimensional space for visualization [28]. The Rtsne function in “Rtsne” package is used to implement the Barnes-Hut tSNE algorithm [28], and here are the parameter values used in tSNE function: perplexity = 30, early exaggeration factor = 12, learning rate = 200, maximum number of iterations = 3000. The parameter perplexity is a guess about the number of close neighbors of each point [29], and typical values are between 5 and 50 [27]. We have considered the intrinsic stochasticity of tSNE algorithm, and use the set.seed function (random number generator in R) when running the algorithm to ensure the results and figures are reproducible. The preliminary results of LAMP classification are objectively presented, and the refined process of the classification results is based on high-dimensional scores, two-dimensional visualization as well as knowledge and experience of diseases. Note that most diseases get closer to diseases of the same category. Focusing on those overlaps helps to discover potential and vital relations between different diseases, which in turn refines the classification.

At the top left of Fig. 6, there are 2 CATG-16 diseases standing close to 15 CATG-10 diseases, listed in Additional file 5(a). All these 17 diseases are related to congenital diseases or disorders of metabolism, with no exception for the two cardiovascular diseases. As mentioned above, most congenital disorders of metabolism are grouped in CATG-10, while CATG-16 contains most congenital malformations, as well as some congenital disorders of metabolism. Inspired by the disease classification in KEGG, specifically, one disease like GM2 gangliosidoses or lysosomal cysteine protease deficiencies can belong to two categories, we refine the disease classification by making the 2 CATG-16 diseases belong to both CATG-16 and CATG-10. In the top of Fig. 6, there are 5 CATG-9 diseases that overlap with 12 CATG-17 diseases, listed in Additional file 5(b). Note that CATG-9 and CATG-17 are adjacent in the top of Fig. 5a, we refine the disease classification by making the 5 CATG-9 diseases mentioned above belong to both CATG-9 and CATG-17, similarly. In the left of Fig. 6, there are 3 CATG-8 diseases that mix up with CATG-5 diseases. Besides, near the main cluster of CATG-5, there are 27 CATG-4 diseases and 12 CATG-5 diseases stand close, which includes two cancers (myelofibrosis and essential thrombocytosis) and two neurological diseases (autism and AD) of concern, listed in Additional file 5(c). We refine the disease classification by making the 3 CATG-8 diseases belong to both CATG-8 and CATG-5, and making the 24 CATG-4 diseases belong to both CATG-4 and CATG-5.

**Fig. 7:**
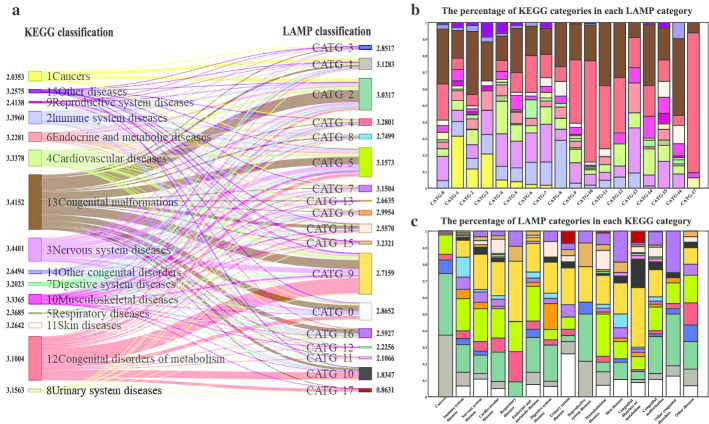
Refined classification results. **a** Illustration of refined diseases classification results. Overall, 15 types of diseases in KEGG are classified into 18 categories. The number next to each category is the entropy that measures the compositional complexity. **b** A stacked bar graph showing the percentage of KEGG categories in each LAMP category. The colors correspond to KEGG categories in **a**. **c** A stacked bar graph showing the percentage of LAMP categories in each KEGG category. The colors correspond to LAMP categories in **a**. The detailed percentages are listed in Additional file 7

Forming a circle in the middle of Fig. 6 are diseases in CATG-1,3,4,6,7,13 and 15. Failing to visualize well-separated homogeneous groups in the two-dimensional tSNE diagram does not necessarily mean that the data cannot be correctly classified. The two dimensions may not be low enough to accurately represent the internal data structure [30]. What those diseases have in common is high impact on more pathways, illustrated in Fig. 5a. Reducing high-dimensional data to two-dimensional data will lose some characteristic information. As the classification model utilizes the full 317-dimensional information for each disease, namely, the normalized impact score vector on pathways, no refined measure is taken for the classification results for these categories. Overall, 15 types of diseases in KEGG are classified into 18 categories illustrated in Fig. 7a, also detailed in Additional file 6.

### Overlapping disease genes unnecessarily lead to the same category in LAMP

Many disease names are related to the locations of the lesions, which may not help identify potential pathogenic mechanisms because diseases with the same lesions may vary widely. Similarly, it makes sense that diseases with overlapping genes may not belong to the same category in LAMP. Table 1 illustrates the over-representation analysis of disease pairs with overlapping gene(s). 1726 diseases make up 1,488,675 pairs, of which 4629 pairs with overlapping gene(s). The number of disease pairs in LAMP and KEGG categories is listed. In Fisher test *p* value < 0.001, which implies that disease pairs with overlapping gene(s) are more likely to belong to the same LAMP or KEGG category. Meanwhile, overlapping disease genes unnecessarily lead to the same category in LAMP and KEGG. In the case of metabolic diseases, Glycogen storage disease (22 genes) and Muscle glycogen storage disease (13 genes) belong to CATG-17 and CATG-10, respectively. Although there are 13 overlapping genes, more genes in Glycogen storage disease lead to impact difference on pathways. In the case of cardiac diseases, there are 11 overlapping genes between Hypertrophic cardiomyopathy (14 genes) and Dilated cardiomyopathy (34 genes), which belong to CATG-2 and CATG-6, respectively. In addition, many disease pairs with overlapping genes are not in the same category in LAMP. The co-occurrence of diseases provides knowledge that can promote drug utilization and develop targeted treatment strategies [31]. For example, mutations in NRAS have been associated with many cancers as well as autoimmune lymphoproliferative syndrome [13]. RET is a proto-oncogene, and mutations in this gene can cause renal agenesis [13]. More attention should be paid to the research of feasible combined treatment in clinical practice.

**Table 1:** Over-representation analysis of disease pairs with overlapping gene(s)

	With overlapping gene(s)	All	Fisher test
In the same LAMP category	1593	170,977	*p* value < 0.001
Not in the same LAMP category	3036	1,317,698	
In the same KEGG category	2707	229,497	*p* value < 0.001
Not in the same KEGG category	1922	125,9178	

1726 diseases make up 1488675 pairs, of which 4629 pairs with overlapping gene(s). The number of disease pairs in LAMP and KEGG categories is listed. In Fisher test *p* value < 0.001, which implies that disease pairs with overlapping gene(s) are more likely to belong to the same LAMP or KEGG category. Meanwhile, overlapping disease genes unnecessarily lead to the same category in LAMP and KEGG

For disease pairs in the same LAMP category but not in the same KEGG category, or in the same KEGG category but not in the same LAMP category, there may be inspiration for treatment. For example, in KEGG, Polycythemia vera (disease gene: JAK2) is a cancer of haematopoietic and lymphoid tissues. Budd-Chiari syndrome (disease gene: F5, JAK2) is Cardiovascular diseases. Both diseases belong to CATG-5 in LAMP. It makes sense because there is evidence that many patients have Budd-Chiari syndrome as a complication of polycythemia vera [32]. In addition, JAK2 is the overlapping gene, and disregulation of the IL6/JAK2/STAT3 signalling pathways can lead to increased cellular proliferation and myeloproliferative neoplasms of hematopoietic stem cells. Besides, Acute myeloid leukemia (AML) is a cancer of the myeloid line of blood cells, characterized by the rapid growth of abnormal cells that build up in the bone marrow and blood and interfere with normal blood cell production. Chronic myeloid leukemia (CML) is a cancer of the white blood cells. It is a form of leukemia characterized by the increased and unregulated growth of myeloid cells in the bone marrow and the accumulation of these cells in the blood. Both AML and CML are Cancers of haematopoietic and lymphoid tissues in KEGG, but they are not in the same LAMP category, because compared with CML, AML has greater impact scores on almost pathways. This result is consistent with the fact that AML is a more intractable disease than CML.

### Entropy of KEGG and LAMP categories: evaluating the prospects for combination diagnosis and gene-targeted therapy

The percentage of KEGG categories in each LAMP category is shown in Fig. 7b, and the percentage of LAMP categories in each KEGG category is shown in Fig. 7c. The detailed percentages are listed in Additional file 7. Here, entropy is introduced to measure the compositional complexity for each category, and shown in Fig. 7a. The lower the entropy, the smaller differences within the category. Conversely, the higher the entropy, the greater the diversity in etiology and pathology within the category. In KEGG classification, the entropy of cancers is the lowest, followed by respiratory diseases and reproductive system diseases. The entropy of nervous system diseases it the highest, followed by congenital malformations and immune system diseases. Figure 7c visually shows support for the results. We can clearly see that the number of LAMP categories in Cancers is smallest, and the LAMP categories in nervous system diseases is rather uneven. In LAMP classification, the entropy of CATG-17 is the lowest, followed by CATG-10,11,12. The entropy of CATG-4 is the highest, followed by CATG-15,5,7. Also, Fig. 7b visually shows support for the results.

Medical conditions are usually defined pathologically or clinically rather than etiologically. Heterogeneous disease in medicine are those medical conditions that have several causes. Given a group of patients with certain disease, it is normal to have more than one cause. Therefore, heterogeneity of disease means multiple possibilities of causes. In particular, cancer heterogeneity has been recognized as an important clinical determinant of patient treatment and prognosis. Cancer heterogeneity researches enable us understand the mechanisms, identify genes truly associated with cancer, and gain insight into development of treatment strategies [33–36]. In this study, the identified disease genes are considered as the representation of the corresponding disease, and the classification results derived from layered assessment on modules and pathways in the human gene network lay stress on the similarity of the outcomes of diseases on pathways. For each component of a category, the impact on pathways is approximate. Entropy is introduced to measure the compositional complexity for each category. Among the 15 KEGG categories, the result that Cancers in KEGG classification possesses the lowest entropy implies that the number of LAMP categories in Cancers is smallest and the outcomes of cancers on pathways are close. It is not conflict with the heterogeneity of cancers, since heterogeneity describes the multiple causes of a group patients with a disease, while entropy describes the compositional complexity for each category, of which diseases with approximate impact on pathways. We suggest looking at diseases from both KEGG and LAMP classification, and integrating genetic and tissue information from the perspective of commonalities in etiology and pathology. In addition, it may be of benefit to explore combination diagnosis of diseases in low entropy categories, and to innovate gene-targeted therapy for diseases in high entropy categories.

## Discussion

The precision medicine initiative has been announced to help innovative personalized care, which integrates efforts of patients, clinicians and researchers [37]. Recent progress has led to an understanding of the effects of gene mutations and makes it possible to study human diseases all at once [3, 38, 39]. There is a key hypothesis in the field of network medicine that one disease phenotype reflects several processes that interact in a complex network [14]. General patterns and correlations of human diseases are not obvious from individual disorder studies. While, the network-based approaches make them discernible [40]. Our goal is to classify diseases integrating modules and pathways in the human gene network, since the integration of human gene networks overcomes limited coverage of genes, and incorporating pathway information into disease classification procedure addresses the challenge of cellular heterogeneity across patients.

In 2019, our research team engaged in research on disease classification and published a paper [3]. The following discusses the method differences between this research and the previous paper. The first difference is in the human gene network. In the previous paper, undirected gene-gene interaction information is obtained from NCBI to construct an undirected human gene network. The largest connected component (LCC) of the human gene network possesses 17,274 genes and 289,913 interactions [3]. In this study, the human gene interaction data is obtained from BioGRID, in which the bait-prey directionality is the basis for constructing a directed human gene network. The LCC of the human gene network possesses 17486 genes and 346,351 interactions. Note that 275,134 human-human gene pairs (equivalent to undirected interactions) are overlapping in the two databases. The second difference is in the definition of influence on pathways by diseases. In the previous paper, summation of the closeness centrality of disease genes within the module is used to weight the access efficiency (AE) from disease genes to module genes [3]. In this study, the inverse average layer summation (IALS) is defined to assess the layered influence of diseases on modules. Considering a gene in the forward layered network, the number of layers is the same as the distance between the gene and disease genes (source nodes), which means the equivalence of definition of AE and IALS. Then the jaccard similarity coefficient (JSC) is used to define the relevance of modules and pathways in the previous paper [3], where only the first-order neighbors are considered. In this study the weighted proportion summation (WPS) of modules in accordance with pathways are defined to assess the layered influence on pathways, considering the influence of multi-order neighbors. Note that WPS can degenerate into JSC when take only layer 0 into account, because genes in layer 0 are exactly the first-order neighbors. In this work, the forward layered network starting from a disease gene is used to assess the layered influence on a module, and module genes exist in almost every layer except layer 0. In the definition of IALS, the smaller the average layer of module genes in the forward layered network, the easier to be accessed by the disease gene. The more disease genes in a module, the more significant role the module plays in developing the disease. The reverse layered network from member genes in a pathway is used to assess the layered influence on the pathway. Note that only genes in layer 0 are pathway genes. In the definition of WPS, the proportion of modules in each layer characterizes the set similarity, and the exponential weight is used since genes in layer 0 are the foremost, followed by genes in layer 1 and so on.

The classifications in the two studies are instructive, although there are some differences in the methods. The diseases which intersect with the LCC are considered. Network-based approach demonstrates the importance of pathway and disease genes, and also illustrates the differences between pathways and diseases. In the previous paper, 1728 diseases are screened out. In undirected human gene network, the perturbation of disease gene status brings a series of feedbacks of reachable genes. The effects of diseases on pathways are assessed in the human gene network, and are emphasized from different perspectives. To classify diseases by the intensity of effects on pathways, the normalized impact score is used to measure the difference in intensity between the pathways, and the greatly affected pathways influence the classification results. To classify diseases by the scope of effects on pathways, the binary impact score is used to mark pathways with a score exceeding the average. In this study, 1726 diseases are considered. Layered networks are introduced to distinguish positions of genes in propagating information from disease genes to pathway genes. Focusing on the layered assessment on modules and pathways, the calculation method of impact score is generalized. As discussed above, IALS is equivalent to AE, and WPS is generated from JSC. After hierarchical clustering, there is a refined process for the classification results. The overlap of diseases between CATG-0,1,2,...,17 in LAMP classification and Group1,2,...,12 in the previous paper can be found in Additional file 8.

The diagnostic markers of various disease states can be identified by analyzing gene expression profiles. Using a classifier on the expression level of the marker gene can predict the disease state of a new patient [4]. Integrating information at the level of functional modules, such as signaling pathways, can overcome the challenge of cellular heterogeneity within tissues and genetic heterogeneity across patients [4, 5]. At the level of molecular expression, estimating pathway activation through member gene expression levels provides a biological interpretation for association of expression profiles and specific states of disease. Lee et al. [4] proposed a method to identify condition-responsive genes (CORGs) and infer pathway activity through the combined expression levels of the CORGs. Estimating pathway activity in a new expression profile to classify disease status has been proved to improve the performance on several disease expression datasets, including tumor necrosis factor (wildtype, mutant), prostate (tumor, normal), acute lymphoblastic leukemia (TEL-AML1, HH), breast cancer (metastatic, non-metastatic), lung cancer (poor prognosis, good prognosis). Su et al. [5] proposed a log-likelihood ratio (LLR) method for probabilistic inference of pathway activities, and applied the method to the classification of breast cancer metastasis, achieving better results than other methods such as CORG [4] and PCA. At the level of human gene network, overlaying more genes and combining gene relationships enable us to increase knowledge of associated pathways and disease etiology. In this study, focusing on the layered assessment on modules and pathways, we propose a disease classification approach. For each of 1726 diseases, given the disease gene(s), we obtain IALS to assess the layered influence on modules, and WPS to assess the layered influences from modules to pathways. Then the normalized impact scores on pathways by disease are evaluate and used for hierarchical clustering and refined process. Diseased tissues and organs as well as congenital factors mainly determined the disease classification in KEGG. In this work, layered assessment on the human gene network, especially pathways, results in the LAMP classification. The goal of LAMP classification is to reposition the understanding of diseases and provide a perspective for studying the etiology of diseases, thereby inspiring researchers to explore diagnosis and therapy strategies.

In this study, network-based approach demonstrates the importance of pathway and disease genes, and also illustrates the differences between pathways and diseases. Diversity in etiology and pathology of most categories of diseases motivates us to study diseases from both KEGG and LAMP classification, and integrate commonalities. Representative diseases of concern, such as cancers, metabolic diseases, mental diseases and congenital diseases, are divided into new groups in LAMP classification, which increases the interpretation of the differences between diseases of the same KEGG group, and also guides to recognize the association between diseases of different KEGG groups.

Disease classification is a progression towards precision medicine with the need for precise patient characterization [41]. Our effort on large number of diseases may lead to widespread discoveries. LAMP classification aims to provide insights for clinical practice and explore combination diagnosis for diseases. Focusing on the layered assessment on modules and pathways, the network-based approaches may enable the progress of drug discovery and reposition [42, 43]. Furthermore, faced with this situation that patients do not respond to treatment, LAMP may motivate clinicians and researchers to try new and complementary strategies [44]. Disease classification also meets public health needs. Comprehensive study of known diseases will help to approach challenges of unknown diseases. Indeed, linking network-based genomic science to patient-oriented science still requires a lot of work. It is important for clinicians to evaluate genomic research as a basis for effective treatment of patients [45].

## Conclusion

In this work, human gene interaction data is collected from BioGRID to construct the human gene network. Disease and pathway genes from KEGG is integrated for layered assessment on modules and pathways. The disease classification model LAMP is developed in which 1726 diseases from KEGG are classified into 18 categories. Within each category, the entropy is introduced to measure the compositional complexity, and to evaluate the prospects for combination diagnosis and gene-targeted therapy for diseases. In KEGG classification, it may be of benefit to explore combination diagnosis for cancers, respiratory diseases and reproductive system diseases. Also, it would reward to explore gene-targeted therapy for nervous system diseases, congenital malformations and immune system diseases. In LAMP classification, combination diagnosis for CATG-17,10,11,12 and gene-targeted therapy for CATG-4,15,5,7 are worth to research. Through KEGG and LAMP classification, we are able to view diseases from the perspective of commonalities in etiology and pathology, which motivates clinicians and researchers to reposition the understanding of diseases and explore diagnosis and therapy strategies.

## Methods

### Data collection

The human gene interaction data is obtained from BioGRID [46]. This download directory (https://downloads.thebiogrid.org/BioGRID/Release-Archive/BIOGRID-3.5.170/) contains the 3.5.170 interaction data release from the BioGRID. This release was compiled on Feb. 25th, 2019 and contains all curated interaction data processed prior to this date. The bait-prey directionality is the basis for constructing a directed human gene network. Totally 374,939 directed interactions of 348,335 gene pairs are curated in 473,480 records, in which 17,562 human genes and 5578 nonhuman genes are involved. Particularly, 17,515 human genes are involved in human-human gene interactions. In this work, 17,515 human genes and 346,377 directed interactions make up the human gene network (HGN). A directed network is called (weakly) connected if replacing all of its directed edges with undirected edges produces a connected (undirected) network. A directed graph is called strongly connected if there is a path in each direction between each pair of nodes of the network. A weakly (strongly) connected component of a directed network is a maximal subgraph that is weakly (strongly) connected. The HGN contains 23 connected components. The largest connected component (LCC) of the HGN possesses 17,486 genes and 346,351 interactions. Moreover, the human gene network contains 5327 strongly connected components. The largest strongly connected component (LSCC) of the HGN possesses 12,179 genes and 310,271 interactions. Genes in LSCC can regulate each other through certain paths. In total, 317 human pathways are selected from KEGG PATHWAY, and summarized into 6 categories [11]. There are 7409 pathway genes, and each gene is associated to 3.6414 pathways on average. Besides, 1758 diseases in 15 types are selected from KEGG DISEASE [11] (https://www.kegg.jp/kegg-bin/get_htext?htext=br08402_gene.keg). There are 3390 disease genes, and each gene is associated to 1.714 pathways on average. 1726 diseases are considered in this work because their genes are in the LCC. Detailed diseases information can be found in Additional file 1. Still, there are some disease genes and pathway genes that are not components of HGN, in other words, no interactions related to these genes have been detected. Confirming the interactions of these genes will be of significance for classification, treatment and prevention of diseases.

### Layered network

In order to show the hierarchical flow of information through the network, we introduced the concept of layered network. There are two modes, forward and reverse, that is, from the perspective of the source nodes and target nodes, respectively. When attaching importance to the effectiveness of receiving information from disease genes (source nodes), we adopt the forward layered network to obtain layered downstream nodes. When emphasizing on the effectiveness of transferring information to pathway genes (target nodes), we adopt the reverse layered network to obtain layered upstream nodes. The forward (reverse) layered network is different according to different source (target) nodes. An example network and specific layered networks are illustrated in Fig. 3a. The layered networks can be obtained by the following algorithm. 
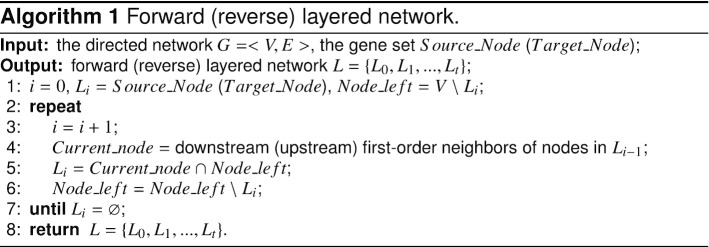


### LAMP: layered assessment on modules and pathways

Figure 3b shows a summary of the LAMP classification approach. Here we introduce the concept of normalized impact score (NIS) of diseases on pathways, which is used to obtain the classification results in the right branch. The number of diseases, modules and pathways are represented by $N_d=1726$, $N_m=11$ and $N_p=317$, respectively. Diseases, modules and pathways are represented by $D_l (l=l,2,...,N_d)$, $M_t (t=1,2,...,N_m)$, $P_k (k=1,2,...,N_p)$. The inverse average layer summation (IALS) of disease genes within modules are defined as follows to assess the layered influence on modules,

$$\begin{aligned} IALS(D_l,M_t) = \sum _{g_i\in D_l}\frac{\sum _{s}\left| M_t\cap F\_layer_s^{g_i}\right| }{\sum _{s} \left| M_t\cap F\_layer_s^{g_i}\right| \times s}\delta (g_i,M_t), \end{aligned}$$

where $F\_layer^{g_i}$ is the forward layered network of gene $g_i$, and $F\_layer_s^{g_i}$ is the set of genes in layer *s*. For $g_i\in M_t$, $\delta (g_i,M_t)=1$, for $g_i\not \in M_t$, $\delta (g_i,M_t)=0$. The weighted proportion summation (WPS) of modules in accordance with pathways are defined as follows to assess the layered influence on pathways,

$$\begin{aligned} WPS(M_t,P_k) = \sum _{s}\frac{\left| M_t\cap R\_layer_s^{P_k}\right| }{\left| R\_layer_s^{P_k}\right| }\times 2^{-s}, \end{aligned}$$

where $R\_layer^{P_k}$ is the reverse layered network of pathway $P_k$, and $R\_layer_s^{P_k}$ is the set of genes in layer *s*. The impact score of disease $D_l$ on pathways is measured by:

$$\begin{aligned} IS(D_l,P_k)= & {} \sum _{t=1}^{N_m} IALS(D_l,M_t) \cdot WPS(M_t,P_k), \quad k=1,2,...,N_p, \\ NIS(D_l,P_k)= & {} \frac{IS(D_l,P_k)-\min _h(IS(D_l,P_h))}{\max _h(IS(D_l,P_h))-\min _h(IS(D_l,P_h))}, \\ NIS(D_l)= & {} (NIS(D_l,P_1),NIS(D_l,P_2),...,NIS(D_l,P_{N_p})). \end{aligned}$$

$NIS(D_l)$ is the normalized impact score vector on pathways by disease $D_l$. The Euclidean distance is used to measure the distance between two diseases. The hierarchical clustering dendrogram of diseases by Ward.D2 method [47] is regarded as a series of partitions. The corresponding difference vector [3] is calculated, in which each element is the difference in average disease distance within and between partitions. The dendrogram is cut where the absolute difference reaches the maximum, then the number of categories is determined [3]. Finally, the entropy is introduced as follows to measure the compositional complexity for each category,

$$\begin{aligned} H = -\sum _{i=1}^{v}p_i \times log_{2}p_i, \end{aligned}$$

where *v* is the number of compositional categories, $p_i$ is the fraction of diseases from category *i*, and $\sum _{i=1}^{v}p_i=1$. Low entropy means small differences within the category, which may be beneficial to explore combination diagnosis of diseases. High entropy means great diversity in etiology and pathology within the category, which may promote the innovation of gene-targeted therapy for diseases.

## Supplementary information

Additional file 1. The 1726 diseases screened in KEGG DISEASE. For each disease, detailed information is listed, including H number in KEGG DISEASE, disease class in KEGG, gene symbol and gene ID.

Additional file 2.The size of modules in each part of LCC. UP$\cup$LSCC is the union of UP_LSCC and LSCC. DOWN$\cup$LSCC is the union of DOWN_LSCC and LSCC. The number in brackets is the number of driver nodes obtained by implementing the maximum matching algorithm.

Additional file 3. The 151 diseases in CATG-0. For each of the 151 CATG-0 diseases, detailed information is listed. All the disease genes are in DOWN_LSCC.

Additional file 4. Preliminary results of 1726 diseases in LAMP classification. Category in preliminary LAMP classification of each diseases is listed.

Additional file 5. Detailed diseases in 2 categories in refined LAMP classification. (a) Information of 15 diseases in CATG-10 and 2 diseases in CATG-16 that stand close at the top left of Fig. 5a. *Denotes that GM2 gangliosidoses appears twice, since the second-level disease classes in KEGG are different. **Denotes that lysosomal cysteine protease deficiencies appears twice, since the first-level disease classes in KEGG are different. (b) Information of 5 diseases in CATG-9 and 12 diseases in CATG-17 overlapping in the top of Fig. 5a. *Denotes that pyruvate dehydrogenase complex deficiency appears twice, since the second-level disease classes in KEGG are different. **Denotes that muscular dystrophy-dystroglycanopathy type B appears twice, since the first-level disease classes in KEGG are different. (c) Information of 3 diseases in CATG-8, 27 diseases in CATG-4 and 12 diseases in CATG-5 that near the main cluster of CATG-5 in the left of Fig. 5a. **Denotes that Atopic dermatitis and Myopathy with lactic acidosis and sideroblastic anaemia appears twice, respectively, since the first-level disease classes in KEGG are different.

Additional file 6. Refined results of 1726 diseases in LAMP classification. Category in refined LAMP classification of each diseases is listed.

Additional file 7. The percentage of KEGG categories in each LAMP category, and the percentage of LAMP categories in each KEGG category.

Additional file 8. The overlap of diseases between CATG-0,1,2,...,17 in this study (LAMP classification) and Group1,2,...,12 in the previous paper (Royal Society Open Science 6(7), 190214 (2019)).

## Data Availability

All data generated or analysed during this study are included in this published article and its supplementary information files.

## Abbreviations

LAMP: layered assessment on modules and pathways
BioGRID: biological general repository for interaction datasets
KEGG: kyoto encyclopedia of genes and genomes
HGN: human gene network
LCC: largest connected component
LSCC: largest strongly connected component
D: disease genes
P: pathway genes
NIS: normalized impact score
AD: Alzheimer’s disease
DAO: D-amino acid oxidase
SNP: single nucleotide polymorphism
tSNE: t-distributed stochastic neighbor embedding
AML: acute myeloid leukemia
CML: chronic myeloid leukemia
AE: access efficiency
IALS: inverse average layer summation
JSC: Jaccard similarity coefficient
WPS: weighted proportion summation
CORG: condition-responsive gene
LLR: log-likelihood ratio
